# Solar Panel Surface Defect and Dust Detection: Deep Learning Approach

**DOI:** 10.3390/jimaging11090287

**Published:** 2025-08-25

**Authors:** Atta Rahman

**Affiliations:** Department of Computer Science, College of Computer Science and Information Technology, Imam Abdulrahman Bin Faisal University, P.O. Box 1982, Dammam 31441, Saudi Arabia; aaurrahman@iau.edu.sa

**Keywords:** solar panel defect detection, computer vision, YOLOv11, deep learning, real-time monitoring, renewable energy, proactive maintenance, Saudi Vision 2030

## Abstract

In recent years, solar energy has emerged as a pillar of sustainable development. However, maintaining panel efficiency under extreme environmental conditions remains a persistent hurdle. This study introduces an automated defect detection pipeline that leverages deep learning and computer vision to identify five standard anomaly classes: Non-Defective, Dust, Defective, Physical Damage, and Snow on photovoltaic surfaces. To build a robust foundation, a heterogeneous dataset of 8973 images was sourced from public repositories and standardized into a uniform labeling scheme. This dataset was then expanded through an aggressive augmentation strategy, including flips, rotations, zooms, and noise injections. A YOLOv11-based model was trained and fine-tuned using both fixed and adaptive learning rate schedules, achieving a mAP@0.5 of 85% and accuracy, recall, and F1-score above 95% when evaluated across diverse lighting and dust scenarios. The optimized model is integrated into an interactive dashboard that processes live camera streams, issues real-time alerts upon defect detection, and supports proactive maintenance scheduling. Comparative evaluations highlight the superiority of this approach over manual inspections and earlier YOLO versions in both precision and inference speed, making it well suited for deployment on edge devices. Automating visual inspection not only reduces labor costs and operational downtime but also enhances the longevity of solar installations. By offering a scalable solution for continuous monitoring, this work contributes to improving the reliability and cost-effectiveness of large-scale solar energy systems.

## 1. Introduction

Solar energy has recently gained significant attention as a potential sustainable and renewable energy source. Solar panels, also known as photovoltaic panels, harvest solar energy from the sun to provide the energy we use every day [1]. Using renewable energy is commonly viewed as contingent upon developing sustainable energy sources, decreasing reliance on fossil fuels, and mitigating climate change [2]. It is crucial for decreasing global warming and reducing greenhouse gas emissions. Furthermore, it can significantly help minimize the amount of water needed to produce energy and improve air quality. However, solar panel installation on land may have adverse effects on nearby species, habitats, soil, and water supplies [3]. When sunlight falls on the solar cells in the panels, the solar energy is absorbed, producing electrical charges. The energy makes the electrons shift within the semiconductor material (usually silicon), creating direct current (DC) electricity. The generated electricity can either be consumed directly or stored in batteries to be used later. Generally, solar panels represent a clean and renewable power source that does not produce greenhouse gases [4]. Consequently, this power may be fetched to DC to Alternating Current (AC) converter (DC2AC) to produce electricity, which can be used to generate power. Although devices and home appliances are available on the market that operate directly on DC, such as lights, fans, and burners, most existing and new devices operate on AC. Solar panels are typically placed in areas with maximum sun exposure, such as rooftops or large solar farms, to provide sustainable energy and reduce reliance on fossil fuels [4]. These panels can either expand the electrical supply of a building or provide electricity in remote or off-grid locations [5].

Dusty solar panels are unable to produce the desired output. Through several investigations at varying dust levels on the solar panels, Chen et al. [6] observed that a dust density of 10 g per square meter decreased the output power of solar panels by 34%. Moreover, photoelectric conversion efficiency and filling factor (FF) also decline with the increase in dust density. They concluded that for every 10 g per square meter rise in dust mass density, the conversion efficiency reduces by 3.4% on average. Solar energy is a significant part of the world’s transition to cleaner power, and Saudi Arabia is investing heavily in solar farms as part of a potential shift away from its reliance on oil-based power. However, harsh weather conditions, such as dust storms, scorching heat, and sudden rain, make panel care particularly challenging. Manual checks are slow and may miss early warning signs. If minor defects are identified promptly, they can be addressed before they impact on the farm’s overall performance. Automating the process means less time walking the fields, fewer missed defects, and more consistent energy production, helping to meet clean energy goals with better reliability and lower costs [6].

Manual inspection, which involves walking among thousands of solar panels or scanning them one by one with thermal cameras, is time-consuming and requires a lot of effort; thus, it is humanly impossible. On large solar farms, defects such as dust covers, small cracks from hail, physical damage from tools or animals, and snow buildup can go unnoticed until the energy output drops sharply. Fixing issues after panels have already lost efficiency leads to wasted electricity and increased costs. A better approach is needed to quickly and reliably spot surface-level problems using regular camera images so that repair teams know exactly where to focus before a minor flaw becomes a significant loss.

Deep learning object detection models, such as YOLOv11, can process images in real time. By training on thousands of panel images labeled with dust spots, cracks, dents, and snow areas, the model learns to spot these issues under varied lighting and angles. Data augmentation, such as flipping images, rotating them, or adjusting brightness before training makes the detector more resilient against real-world conditions. Once trained, the model runs on small, inexpensive computers right at the site. Clear labels for each defect type enable technicians to identify what requires urgent repair and what can wait for routine cleaning.

### 1.1. Related Work

#### 1.1.1. Computer Vision Approaches for Solar Panel Defect Detection

Recent advances in computer vision and deep learning have enabled significant progress in automated solar panel defect detection. This section reviews key studies that have contributed to this rapidly evolving field.

Zhang and Yin [7] developed an improved YOLOv5 algorithm for identifying solar cell surface defects. Their model incorporates deformable convolution and attention mechanisms to adaptively scale defect detection based on feature size. Through enhancements in data augmentation and feature extraction techniques, their approach achieved a mean average precision (mAP) of 89.64%. Resultantly outperforming earlier versions in both precision and speed, making it particularly suitable for photovoltaic monitoring applications. Advancements in attention-based mechanisms have further improved detection accuracy. Dwivedi et al. [8] proposed a Vision Transformer (ViT) model for detecting surface defects in renewable energy assets, including solar panels. This approach demonstrated superior performance compared to traditional architectures like ResNet50 and MobileNet across various environmental conditions, highlighting its potential for large-scale solar farm monitoring.

#### 1.1.2. Specialized Deep Learning Models for Defect Classification

Researchers have developed increasingly specialized models to address the unique challenges of solar panel defect detection. Prabhakaran et al. [9] created a Multi-Variant Deep Learning Model (RMVDM) that employs advanced preprocessing techniques, including Region-Based Histogram Approximation (RHA) and Gray Scale Quantization Algorithm (GSQA). These methods significantly enhance image processing workflows, facilitating more accurate and efficient defect detection on solar panel surfaces.

Al-Otum [10] developed a sophisticated deep learning system for defect detection using electroluminescence (EL) images. The Independent Light-Depth Convolutional Neural Network (CNN-ILD) model features multiple branches, each analyzing different image aspects to capture important details. This approach achieved accuracy between 88.41% and 98.05% in identifying defects on solar panels, demonstrating considerable effectiveness compared to similar models.

Shao et al. [11] introduced a novel method for detecting dust on photovoltaic panel surfaces based on deep learning. A new, enhanced Adam optimization algorithm is proposed in this work for detecting surface dust on solar photovoltaic panels. While the classical Adam algorithm is the preferred choice for optimizing neural network models, it sometimes encounters issues such as local optima, overfitting, and non-convergence due to inconsistencies in the learning rate during the optimization process. To counter these challenges, the enhanced algorithm integrates Warmup technology and cosine annealing techniques with the conventional Adam algorithm, providing a gradual adjustment of the learning rate to maintain stability during the initial stages of training. Herein, they found enhanced performance for dust detection in three deep learning models, such as ResNet-18, VGG-16, and MobileNetV2.

#### 1.1.3. YOLO-Based Approaches for Real-Time Applications

YOLO (You Only Look Once) algorithms have proven remarkably successful in real-time defect detection applications. Haeruman et al. [12] utilized YOLOv7 to detect defects in PV panels by analyzing infrared images captured by drones. Their model achieved a paramount average precision of 85.9%, making it well suited for real-time deployment in solar farm environments. Building on this foundation, Cao et al. [13] developed an advanced YOLOv8-GD model for detecting defects in solar panels using electroluminescence images. By enhancing feature extraction procedures and integrating DW-Conv (DepthWise Convolution) into the YOLOv8 backbone, they achieved a mean average precision (mAP) of 92.8% at an IoU threshold of 0.5 and 63.1% from 0.5 to 0.95. This significant improvement in accuracy, coupled with a 16.7% reduction in model size, demonstrates YOLOv8-GD’s potential for efficient real-time PV panel inspection. Özer and Türkmen [14] proposed a deep learning approach for detecting the condition of solar panels in solar plants. In this regard, they have investigated various members of the YOLO family, including YOLOv5, YOLOv7, and YOLOv8 for varying numbers of epochs. Consequently, they found that YOLOv5 exhibited the highest F1-score value at 150 epochs, which was 97%. They aimed to deploy the model using a drone for periodic inspection of solar panels at the power plant. It is commonly observed that the YOLO family has been most widely used in solar panel defect detection, especially YOLOv5 and YOLOv8 [15].

Like solar panel defect detection, the YOLO family has various applications in related areas, such as printed circuit board (PCB) defect detection proposed by Mo et al. [16]. The authors investigated a YOLOv5s-based improved model, coined as SE-ENv2 GC-Neck TSCODE (SGT-YOLO), to provide a better trade-off between model complexity and accuracy. The proposed approach improved the mAP and mAP0.5 by 2.7% and 6.4% on a state-of-the-art PCB defect detection dataset. Similarly, the YOLO family has been successfully employed for insulator defect detection in transmission lines [17] and Unmanned Aerial Vehicles (UAV) imagery-based fire detection [18].

#### 1.1.4. Fully Automated Systems and Alternative Approaches

Bartler et al. [19] designed a fully automated system for detecting defects in solar cells using electroluminescence images. Their process begins with image cleaning and distortion correction before isolating areas of interest. A deep CNN then classifies various defect types, including cracks, dislocations, and discolorations. To address limited training data, they employed data augmentation techniques to create additional examples and improve model reliability. Zhang and Duranay [20] explored an alternative approach using infrared solar module images for defect classification. Their EfficientNetB0 model combined with a support vector machine (SVM) classifier achieved 93.93% accuracy in classifying defects across 12 different classes, demonstrating the effectiveness of hybrid deep learning approaches in enhancing solar energy system performance.

Akin to solar panel defect detection, there are several applications of deep learning algorithms in similar types of object detection in industrial and related settings. For instance, Sun et al. [21] presented a novel multi-scale attention network for insulator detection in a foggy environment. The authors claimed that the proposed approach was promising compared to previous approaches in various aspects. Similarly, UAVs have been most widely used for a variety of applications, including flame detection in forest fires. This can help in timely intervention and prevention of unwanted fire incidents. In this regard, Ren et al. [22] proposed a Strong Saliency Features Guide Subnetwork (SSFGS) for flame detection. The proposed scheme outperformed the state-of-the-art on a benchmark dataset with a recall and mAP-50 of 79.2% and 82.4%, respectively.

Table 1 presents a summary of related work. Based on the summary, it is apparent that solar panel defect detection and dust detection are two separate areas of research, and the studies mainly focused on a single aspect of the problem.

**Figure 1:**
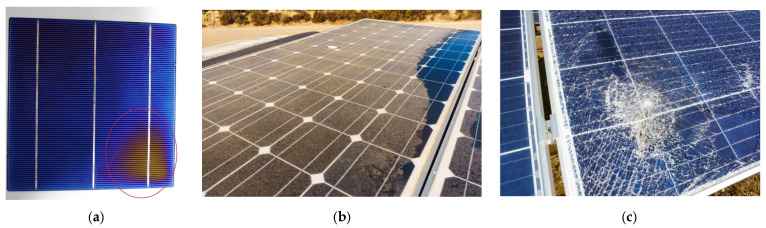
(**a**) Defective, (**b**) dusty panel, (**c**) physical damage.

As far as the defect detection is concerned, it usually covers burns, hotspots, and solar cell damage as depicted in Figure 1a. In terms of dust detection, it mainly covers the sand and dust covering the solar panels that may hinder the energy conversion process as illustrated in Figure 1b which is a major case in Saudi Arabia due to heavy and frequent sandstorms. Likewise, physical damage is depicted in Figure 1c.

To address this aforementioned gap, the proposed study presents a comprehensive model for detection of solar panel defects, dust, and snow at the same time. This results in a multi-class classification problem with five classes including Non-Defective, Dust, Defective, Physical Damage, and Snow on photovoltaic surfaces. In this regard, various datasets from open sources have been integrated and augmented for the development of a proposed YOLOv11-m model.

The rest of the paper is organized as follows: Section 2 presents the proposed methodology; Section 3 presents the experimental results; Section 4 is dedicated for discussion, while Section 5 concludes the study.

## 2. Materials and Methods

This section presents the proposed methodology for real-time monitoring of solar panel health across five classes: Non-Defective, Dust, Defective, Physical Damage, and Snow. The workflow combined multi-source imagery, unified it under a single annotation schema, and trained a YOLOv11-based detector.

**Figure 2:**
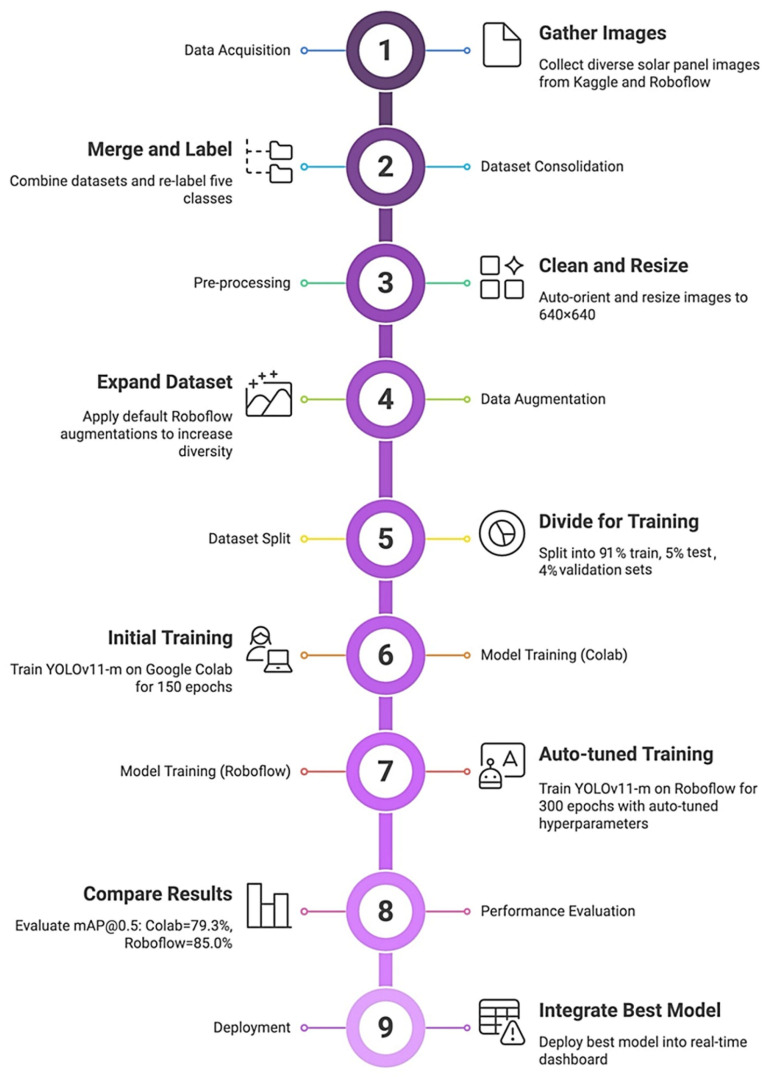
Solar panel model workflow.

Figure 2 presents the methodological workflow of the proposed solar panel dust and defect detection model, starting with data collection, labeling, and consolidation of the dataset. Consequently, the dataset is pre-processed through cleansing and resizing. Data augmentation is performed afterwards to make the proposed models robust and generalized. Model development, training, and testing are then accomplished in Google Colab, and the model is subsequently fine-tuned in RoboFlow. Model evaluation and comparison with the state-of-the-art is performed, and finally, it is deployed and integrated into the software prototype.

### 2.1. Methodology

#### 2.1.1. Data Acquisition

To develop a robust and multi-class solar panel dust and defect detection model, a diverse dataset has been developed from various open sources. The details of each open source are as follows:

- Dust images: Kaggle “Solar-Panel Dust Detection” [23]. Containing two classes, dusty (1493) and clean (1069), with a total of 2562 instances.
- Non-Defective, Physical Damage, Snow: Roboflow “Solar-Panel Detection” project [24]. Containing a total of 2642 images.
- Defective, Snow supplements: Roboflow “6Rainstorm Final Project” [25]. Containing a total of 3640 images.

The following interpretation was assumed:

1. Defective (cracks, hotspots, cell damage).
2. Dust (obstructing the panel surface including dust and bird drop, bird feather etc.).
3. Non-Defective (clear and no observable defects).
4. Physical Damage (frame deformation, broken glass, impact damage).
5. Snow (snow accumulation reducing panel efficiency).

#### 2.1.2. Dataset Consolidation

The final solar panel corpus was built by cherry-picking the highest-quality images from three public collections as mentioned above—Kaggle’s Solar-Panel Dust Detection (for dust-laden surfaces), Roboflow’s Solar-Panel Detection project (providing clean modules, physical damage shots, and snow scenes), and the Roboflow 6Rainstorm Final Project (adding additional defective and snow instances). In this regard, the relevant details are provided in Table 2.

All raw annotations were remapped into a unified five-class scheme—Non-Defective, Dust, Defective, Physical Damage, Snow—and duplicate or low-resolution frames were discarded. The merger produced 8973 RGB best-quality images covering a wide range of illumination, camera angles, and environmental conditions typical of large-scale photovoltaic farms before augmentation procedures.

Before training, every image was auto-oriented and letter-boxed to a fixed 640 × 640 resolution, then partitioned into 8212 training images, 440 test images, and 321 validation images. Eventually, the labels were exported to YOLO-txt format to ensure compatibility with the Ultralytics pipeline. The aggressive augmentation policy (flips, multi-angle rotations, zoom-crops, shear, grayscale injection, color jitter, and pixel noise) generated three synthetic variants per original image, enriching minority classes and improving robustness to dust haze, lens glare, and seasonal artifacts.

#### 2.1.3. Data Pre-Processing and Augmentation

To build a robust model capable of fine detection across various environmental and angular conditions, the following augmentations have been employed. The range of each type of augmentation has also been provided. The selection of augmentation types and their values is based on the literature review and the most widely targeted weather and environmental conditions.

- Flips: horizontal and vertical.
- 90° rotations (CW and CCW).
- Random zoom-crop (0–20%).
- Free rotations ±15°.
- Shear ±10% (H and V).
- Grayscale on 15% of images.
- Saturation ±20%, exposure ±10%.
- Gaussian noise ≤ 0.3% pixels.
- Auto-orient → resize to 640 × 640 (letter-boxed).

#### 2.1.4. Dataset Cleaning, Normalization, and Scaling

Pixel values are normalized to improve convergence during training. Whereas scaling ensures consistency in object size representation across the dataset. Annotation was performed using a combination of Grounding DINO and manual labeling (refined via Roboflow), ensuring proper bounding box placement and consistent labeling across all images [26]. Additionally, the following operations were performed on the dataset: removing irrelevant or unusable images; the images that do not match the intended use case or are of poor quality were discarded.

#### 2.1.5. Model Training Environments

For model building, training, testing, and validation, two state-of-the-art environments have been considered as follows:

1. Google Colab: YOLOv11-m backbone, 150 epochs, batch size 32.
2. Roboflow Train: The same dataset, with Roboflow’s hyperparameter sweeps and early stopping. Highest-accuracy Roboflow weights integrated into our existing dashboard for live inference and alerting.

#### 2.1.6. Model Deployment

Finally, the trained model was deployed and validated through real-time webcam integration tests, ensuring robustness before deployment in an industrial setting. For the final model training phase, Google Colab was utilized, leveraging its powerful computing resources. Technical details of the final model include: Ultralytics Version: 8.3.40, Python: 3.11.11, Torch: 2.6.0+cu124, CUDA:0 (NVIDIA L4, 22693MiB), and YOLOv11s summary (fused): 238 layers, 9,416,283 parameters, 0 gradients, and 21.3 giga floating point operations per section (GFLOPs).

### 2.2. Proposed YOLOv11-Based Approach

For the research, the YOLOv11-m architecture is implemented, chosen based on a balance of accuracy and edge-device latency suitable for complex environments [27]. To optimize model performance, meticulous hyperparameter tuning was performed, accompanied by strategic pre-processing and augmentation methods. Data pre-processing involved automatically orienting images and resizing them to a consistent resolution of 640 × 640 pixels.

**Figure 3:**
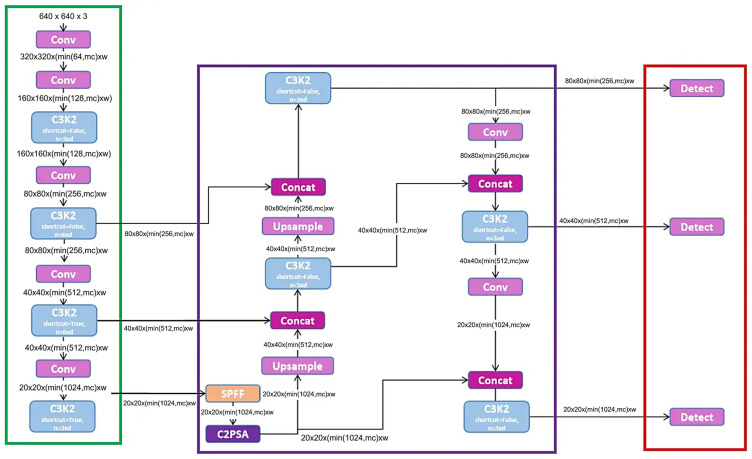
YOLOv11-m default model architecture.

Augmentation strategies significantly enriched the dataset variability as mentioned above. This structured augmentation protocol substantially increased the robustness and generalizability of the detection model, addressing potential variability within real-world environmental conditions. Figure 3 presents the architecture of YOLOv11-m exploited in the study.

### 2.3. Performance Evaluation

The primary metric for evaluating the model’s performance was mean average precision (mAP). Specifically, we focused on the commonly used threshold of mAP@0.5 to align with industry standards and benchmark comparisons [28]. The dataset was strategically split into training, testing, and validation subsets to ensure a balanced evaluation framework. Throughout model training, performance metrics were systematically monitored and logged, allowing iterative refinements via further hyperparameter adjustments and dataset balancing as required. The following formula shows how to calculate the mean average precision:

(1) $$mAP=\frac{1}{N}\sum_{i=1}^{N}{AP}_{i}$$

where *N* is the total number of queries and *AP_i_* is the average precision at *ith* query found by averaging realized precision at each relevant point at the ordered list of outcomes.

Other than that, the accuracy, precision, recall, and F1-score are the most common metrics used for evaluating machine learning and deep learning models as given in Equations (1)–(5), respectively [29,30]. Here the values correspond to true positive (TP), true negative (TN), false positive (FP), and false negative (FN).

(2) $$Accuracy=\frac{TP+TN}{TP+TN+FP+FN}$$

(3) $$Precision=\frac{TP}{TP+FP}$$

(4) $$Recall=\frac{TP}{\left(TP+FN\right)}$$

(5) $$F1\;score=2\times \frac{precision\times recall}{precision+recall}$$

## 3. Results

The final solar panel corpus was built by cherry-picking the highest-quality images from three public collections—Kaggle’s Solar-Panel Dust Detection (for dust-laden surfaces), RoboFlow’s Solar-Panel Detection project (providing clean modules, physical damage shots, and snow scenes), and the Roboflow 6Rainstorm Final Project (adding additional defective and snow instances). All raw annotations were remapped into a unified five-class scheme—Non-Defective, Dust, Defective, Physical Damage, Snow—and duplicate or low-resolution frames were discarded. The merger produced 8973 RGB images covering a wide range of illumination, camera angles, and environmental conditions typical of large-scale photovoltaic farms. Before training, every image was auto-oriented and letter-boxed to a fixed 640 × 640 resolution, then partitioned into 8212 training, 440 test, and 321 validation images. Labels were exported in YOLO-txt format to ensure compatibility with the Ultralytics pipeline, and the aggressive augmentation policy (flips, multi-angle rotations, zoom-crops, shear, grayscale injection, color jitter, and pixel noise) generated three synthetic variants per original, enriching minority classes and improving robustness to dust haze, lens glare, and seasonal artifacts.

**Figure 4:**
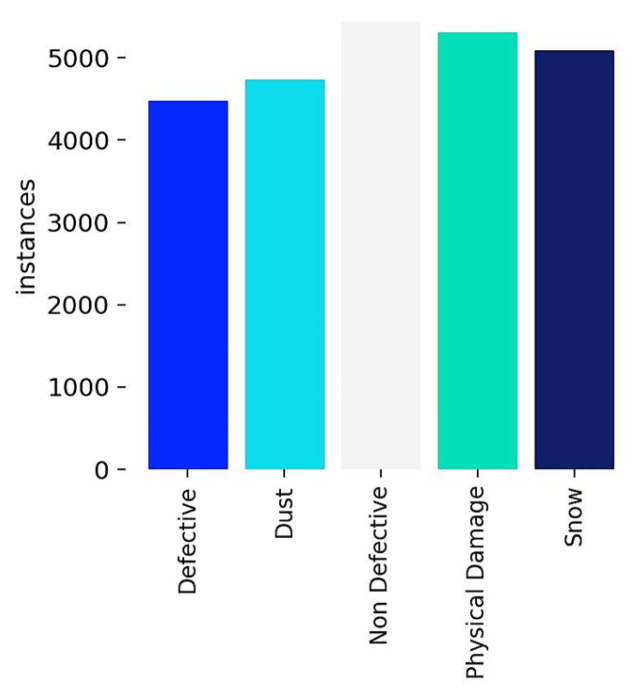
Dataset class distribution.

Figure 4 presents the distribution of instances concerning each label. It is worth noting that the dataset is balanced after the augmentation process is performed, with an enhanced number of around 25,000 images. Nonetheless, Non-Defective, Physical Damage, and Snow classes have moderately more instances than Dust and Defective classes.

### Performance Evaluation of the Proposed Models

Table 3 presents the performance of the proposed solar panel dust and defect detection. The results are obtained for each class, as well as the mean average precision of all the classes. Two training platforms have been utilized for model development and analyses: Google Colab and Roboflow. In terms of speed, Google Colab was faster, while in terms of performance, RoboFlow provided better results.

According to the results, for 115 epochs (optimized) of the Google Colab platform, the proposed scheme exhibited an average mAP@0.5 score of 79.3%. While the Non-Defective class exhibited the highest percentage of 96.6%, followed by the Snow class at 91.1%. The Defective and Physical Damage classes were at 75.7% and 79.5%, respectively. While the Dust class exhibited the poorest performance at 53.7%.

Likewise, in the results for 300 epochs (optimized) in the RoboFlow platform, the proposed scheme exhibited an average mAP@0.5 score of 85%. While the Non-Defective class exhibited the highest percentage of 96%, followed by the Snow class at 90%. The Defective and Physical Damage classes were at 84% and 83%, respectively. While the Dust class exhibited a relatively poor performance at 73%.

Most of the results, including mAP@0.5, are better than those of the Colab version of the proposed model. This is mainly because the optimization of hyperparameters is better performed in RoboFlow, in contrast to Colab; nonetheless, it required more than twice the number of epochs as shown in Table 3.

**Figure 5:**
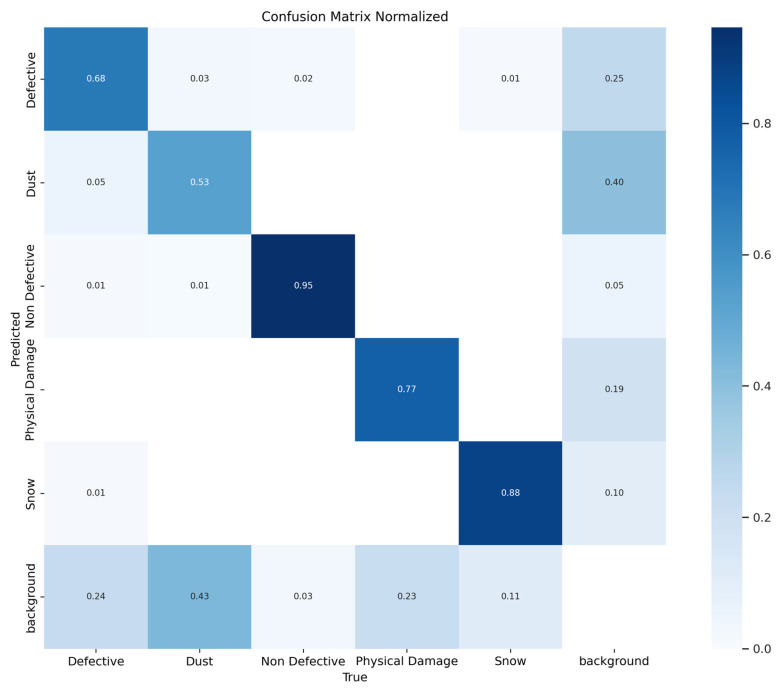
Normalized confusion matrix.

Figure 5 presents the normalized confusion matrix of the proposed model. It is apparent that the classes with best to worst performance, respectively, are Non-Defective, Snow, Physical Damage, Defective, and Dust.

Furthermore, it is interesting to note that if we consider only the Defective and Non-Defective classes, ignoring the rest of the classes (a typical case in binary classification of solar panel defect detection [1,5]). The proposed YOLOv11 model achieves a mAP@0.5 of 90%, which is exceptionally comparable to and improved upon many state-of-the-art studies [1] with a mAP@0.5 of 89.64% and [5] with a mAP@0.5 of 85.9% for binary classification problems.

Moreover, the values of other metrics of the proposed model are given in Table 4 for binary classification. The results are promising in terms of accuracy, precision, recall, and F1-score, all of which exceed 95%.

## 4. Discussion

**Figure 6:**
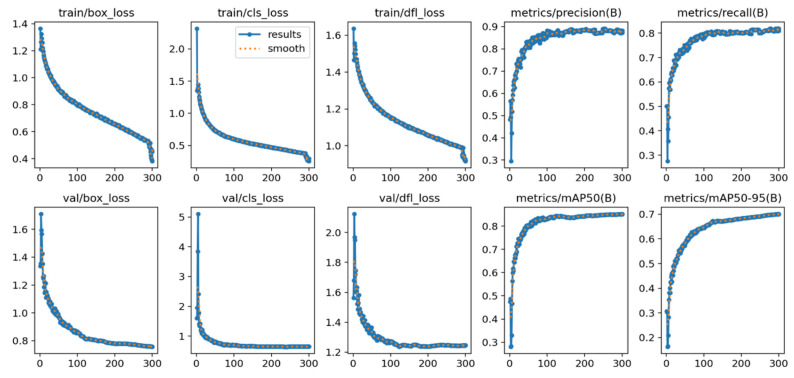
Advanced training graphs.

Figure 6 illustrates the training dynamics of the YOLOv11 model over 300 epochs. The top row tracks training loss components—bounding-box regression (train/box_loss), classification (train/cls_loss), and distribution-focal loss (train/dfl_loss)—all of which fall steeply within the first 50 epochs and continue a smooth, monotonic decline, signaling stable convergence. The corresponding validation losses (bottom row) mirror this trajectory and level off at comparable magnitudes, indicating minimal over-fitting and good generalization to unseen data.

Performance metrics on the same graph confirm this aforementioned trend. Precision climbs rapidly to ≈ 0.88 and plateaus after epoch 120, while recall stabilizes near 0.80, reflecting a balanced detector that captures most defects without an excessive false-positive rate. The overall mAP@0.50 reaches 0.85 by epoch 150 and remains steady, matching the Roboflow evaluation reported earlier. A gradual rise in mAP@0.50–0.95 to ~0.70 demonstrates the model’s competence across stricter IoU thresholds, crucial for pinpointing small cracks or dust patches on panel surfaces.

Collectively, these curves verify that the augmentation regimen and automated hyperparameter tuning achieved consistent optimization without divergence between training and validation sets. The final checkpoint, therefore, meets the stated deployment criterion (≥85% mAP@0.50) and is suitable for real-time solar panel health monitoring.

By replacing routine manual checks on the mounted solar panels, the proposed system aims to free technicians to work on more complex tasks while ensuring panels stay clean and undamaged. Technicians will need training to trust and use AI alerts correctly. Using camera streams raises questions about privacy—images must be stored and handled following local laws. It is also important to explain what the system can and cannot do so users understand that hidden faults still need human inspection. With clear rules and open communication, automated inspection can improve solar energy output safely, and effectively while keeping human expertise at the center of decision-making.

**Figure 7:**
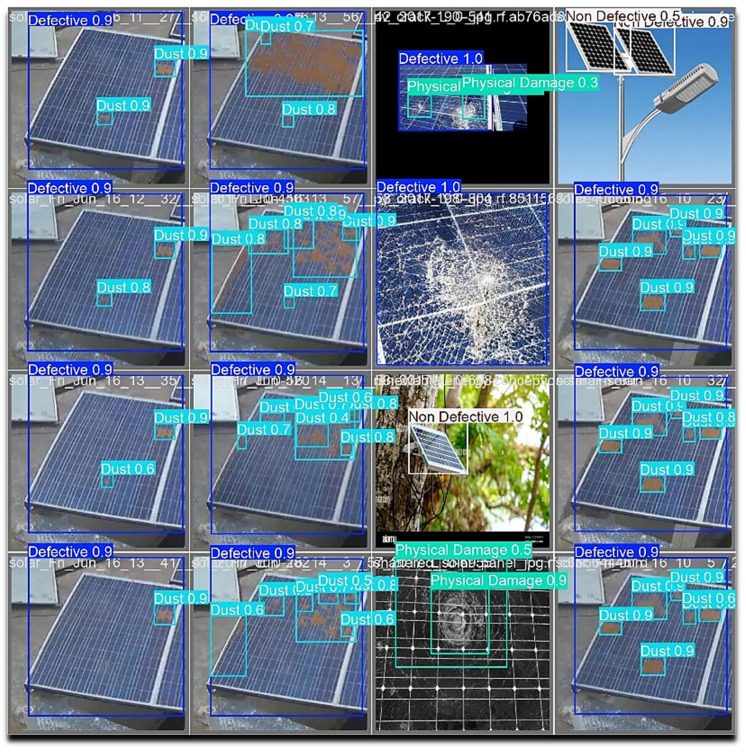
Model’s detection output.

Figure 7 provides the proposed model’s output for various classes. It is apparent that in most cases, the detection accuracy is above 90% which demonstrates the promising nature of the proposed approach for solar panel dust and defect detection for diverse environmental conditions.

**Figure 8:**
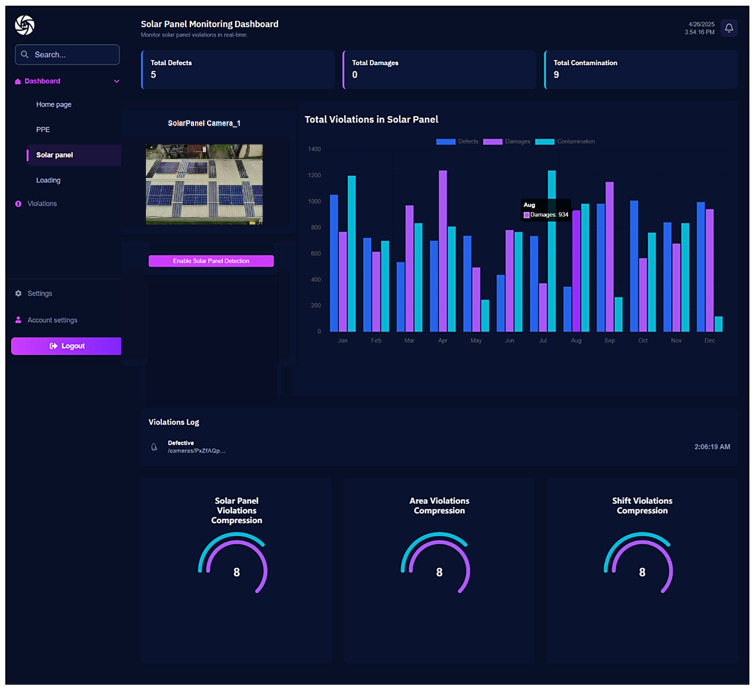
Solar panel inspection dashboard.

Additionally, a software prototype has been implemented to demonstrate the real-time application of the proposed solar panel dust and defect detection model. In this regard, Figure 8 shows the live totals of defects, damage, and contamination in solar panels. It further demonstrates the performance of the proposed system with a live stream captured by the attached camera (solar panel camera-1).

It also provides per-camera detection toggles, plots monthly violation trends in a stacked bar chart, streams a real-time violations log, and summarizes key metrics with gauge widgets. The software accepts input from live cameras or allows users to upload a video to the system. It then breaks down the video frames and fetches them to the trained YOLOv11 model for solar panel dust and defect detection. Consequently, the issues (named as violations in the software) are identified and observed over time, and visual reports are generated. After that, remedial actions can be taken based on the reports generated by the dashboard.

### Limitations of the Study

Overall, the study is promising in its ability to detect solar panel defects and dust in real time accurately. Nonetheless, the proposed research has several assumptions and limitations, which are outlined below.

This study examines only what is visible on the panel surface in normal camera images. It does not detect internal faults, such as short circuits or hot spots, that require thermal scans. The tests were conducted using publicly available photos and field samples under conditions like those in Saudi Arabia’s Eastern Province. Results may differ for panels with unusual coatings, different colors, or under very low or artificial lighting conditions. The system focuses on standard crystalline silicon panels and might not work the same on newer panel types. Regarding the limitations of the study, for a multi-classification problem involving five classes, a significantly larger and more comprehensive dataset is required. Moreover, finer classification can be achieved by introducing additional classes, such as bird droppings, bird feathers, and leaves, for improved classification. As future work, the following steps are to (1) elevate dust detection through targeted image collection and synthetic haze augmentation, (2) add thermal or hyperspectral inputs to reveal subsurface defects, and (3) compress the model to INT8 TensorRT for efficient, on-device inference on Jetson Orin gateways. Additionally, the current study investigated YOLOv11-m as the object detection model; future studies may investigate vision transformers and other members of the YOLO family, such as YOLOv12 (currently in experimental phase). Moreover, to further fine-tune the results with multi-classification, vision transformers can be investigated [31,32].

## 5. Conclusions

The proposed study investigates YOLOv11 for real-time defect and dust detection on solar panels. The study combines two binary classification problems (defective/non-defective) and (dust/clean) into a single multi-class problem. The proposed model addresses a multi-classification problem with five classes, yielding reasonable and acceptable performance. Demonstrating reliable field-readiness, the solar panel model achieves an 85% mAP@0.50 score while maintaining high accuracy, F1-score, and recall above 95% across 300 training epochs. In terms of the application areas of the proposed study, the administration can incorporate the system in smart cities, smart industries, and smart agriculture for automated defects and dust detection of solar panels mounted in remote areas using drones equipped with surveillance cameras. The authorities can then employ smart maintenance measures in response. In the future, researchers can incorporate more data augmentation approaches to cover a wider range of conditions for solar panels. Moreover, other deep learning models, particularly vision transformers, can be investigated to achieve more robust and generalized outcomes.

## Figures and Tables

**Table 1:** Summary of related work.

Ref.	Author/s	Problem	Technique/s	Results
[7]	Zhang and Yin (2022)	Solar cell surface defect detection	Improved YOLOv5, deformable convolution, attention mechanisms	mAP: 89.64%, improved precision and speed for solar cell defect detection
[8]	Dwivedi, et al. (2024)	Identification of surface defects on solar PV panels and wind turbine blades	Attention-based deep learning, vision transformers (ViT)	High performance in detecting defects on solar panels and wind turbine blades
[9]	Prabhakaran, et al. (2023)	Defect detection and localization on photovoltaic panels	Deep learning, pre-processing techniques (RHA, GSQA)	Improved image processing for efficient defect detection
[10]	Al-Otum (2023)	Defect classification in electroluminescence images of solar panels	CNN-ILD (Convolutional Neural Network Independent Light-Depth)	Accuracy between 88.41% and 98.05% for defect classification
[11]	Shao et al. (2024)	Dust detection on solar panels	ResNet-18, VGG-16, and MobileNetV2	Accuracy ResNet-18: 95.12%; VGG-16: 61.21% MobileNetV2: 99.43%
[12]	Haeruman, et al. (2024)	Solar panels inspection	Advanced YOLO algorithm, infrared images	Main average precision: 85.9%, suitable for real-time solar farm inspection
[13]	Cao, et al. (2024)	Defect detection in electroluminescence images of solar panels	Improved YOLOv8-GD, electroluminescence images, DW-Conv	mAP: 92.8%, 16.7% reduction in model size, real-time inspection capability
[14]	Özer and Türkmen (2024)	Detection of solar panel condition in solar plants	YOLOv5, YOLOv7, and YOLOv8	F1-score as 90% to 97%
[20]	Zhang and Duranay (2023)	Fault detection in solar energy systems: a deep learning approach	EfficientNetB0, support vector machine (SVM)	Accuracy: 93.93%, effective in classifying 12 different types of PV defects

**Table 2:** Dataset sources and instances.

#	Dataset	Instances	Actual Classes	Derived Classes
1	Kaggle “Solar-Panel Dust Detection”	2562	Dusty, clean	Dusty, clean
2	Roboflow “Solar-Panel Detection”	2642	Bird drop, bird feather, clean, defective, snow, dusty, leaf	Clean modules, physical damage shots, and snow scenes
3	Roboflow 6Rainstorm Final Project	3460	Bird drop, defective, non-defective, dusty, clean, physical damage	Defective and snowy instances

**Table 3:** Performance comparison with two platforms.

Training Platform	mAP@0.5 (All)	Defective	Dust	Non-Defective	Physical Damage	Snow
Colab (115 epochs)	0.793	0.757	0.537	0.966	0.795	0.911
Roboflow (300 epochs)	0.850	0.840	0.730	0.960	0.830	0.900

**Table 4:** Performance results.

Model	Accuracy	Precision	Recall	F1-Score	Misclassification Rate
YOLOv11-m	95.98%	95.53%	95.53%	95.54%	4.02%

## Data Availability

The dataset obtained for the current study is available on the public repositories Kaggle and RoboFlow.
